# Antigen-specific Th1 cytokine markers and protection against tuberculosis: a systematic review and meta-analysis stratified by progression to active disease and sustained IGRA conversion

**DOI:** 10.3389/fcimb.2026.1780600

**Published:** 2026-02-20

**Authors:** TianYu Lin, Sheng Liu, Yan-Yu Pan

**Affiliations:** 1 Fuzong Clinical Medical College of Fujian Medical University, Fuzhou, China; 2 The Second Department of Infection, 900th Hospital of PLA Joint Logistic Support Force, Fuzhou, China

**Keywords:** correlate of protection, correlate of risk, IFN-γ, IGRA conversion, IL-2, polyfunctional T cells, systematic review, TNF-α

## Abstract

**Background:**

Tuberculosis (TB) remains a leading global cause of infectious mortality. Accelerating vaccine development requires validated immune correlates of protection (CoPs). Mechanistic studies have long highlighted Th1 cytokines (IFN-γ, IL-2, TNF-α) as crucial for anti-mycobacterial immunity, leading to the hypothesis that antigen-specific Th1 responses, particularly polyfunctional T cells, may serve as a CoP. However, clinical evidence linking these responses to protection has been inconsistent.

**Methods:**

We conducted a systematic review and meta-analysis to evaluate antigen-specific IFN-γ, IL-2, and TNF-α as correlates of protection or risk. We searched PubMed/MEDLINE, Embase, Web of Science, and Cochrane Central up to June 30, 2025. We included human studies with longitudinal follow-up that measured these cytokines and reported progression to active TB disease (primary analysis) or sustained IGRA conversion (secondary analysis). Study selection, data extraction, and risk-of-bias assessment were performed in duplicate. Random-effects meta-analyses were conducted where feasible, pooling across different antigen classes (e.g., PPD, BCG, ESAT-6/CFP-10, vaccine antigens) and assay platforms (e.g., ICS, ELISpot, whole-blood). Although these assays and antigens differ in their ability to activate distinct immune responses, the pooled estimates reflect general trends in immune markers across various immunological contexts. This pooling approach was taken to maximize available data and evaluate broad immune responses. Where possible, we conducted subgroup and sensitivity analyses to explore the robustness of the findings across antigen and assay categories.

**Results:**

From 1, 268 records, 10 studies were included. In the primary analysis of active TB disease (n=6 studies), pooled odds ratios for IFN-γ, IL-2, TNF-α, and polyfunctional responses were all close to 1.0 (range: 0.97–1.11) with confidence intervals spanning the null and low heterogeneity (I² = 0%). In the secondary analysis of sustained IGRA conversion (n=5 studies), continuous measures of IFN-γ and IL-2 were marginally higher in converters (pooled MDs: 0.07 and 0.06, respectively). Binary analyses showed a consistent but modest trend toward positive association (pooled ORs: 1.13 for IFN-γ, 1.07 for IL-2), though confidence intervals included 1.0. Sensitivity and subgroup analyses were conducted, including stratification by antigen type (e.g., PPD, BCG, ESAT-6/CFP-10, vaccine antigens) and assay platform (e.g., ICS, ELISpot, whole-blood). While no significant effect modification was observed, this stratification underscores the importance of considering heterogeneity across different antigenic stimuli and assay methodologies when interpreting the pooled results. A Baujat plot identified specific studies (e.g., Kagina et al., Nemes et al.) as primary contributors to heterogeneity.

**Conclusion:**

Available prospective evidence does not support antigen-specific Th1 cytokine magnitudes—individually or as polyfunctional profiles—as reliable, standalone correlates of protection against progression to active TB disease. These responses appear more strongly associated with immune activation states linked to recent antigen exposure or infection risk. The findings underscore the need to look beyond peripheral Th1 cytokine levels, recognizing that the pooled estimates across different antigen types and assay platforms reflect general directional trends rather than directly comparable quantitative effects. This highlights the need for a more nuanced approach, considering the diversity of immune responses induced by varying antigens and assay techniques. Future studies should aim for standardized, harmonized assays and endpoint definitions to allow for more accurate comparisons across different study designs and populations.

**Systematic review registration:**

https://www.crd.york.ac.uk/prospero/, identifier INPLASY202610094.

## 1 Introduction

Tuberculosis (TB) continues to be a major cause of death from infectious diseases globally. In vaccine and immune response studies, the terms ‘correlates of protection’ (CoP) and ‘correlates of risk’ are often used. A correlate of protection refers to a biological marker or immune response reliably associated with a reduced risk of disease, indicating protective immunity. In contrast, a correlate of risk refers to immune markers that may reflect a heightened susceptibility to infection or disease, often linked with recent or ongoing antigen exposure. This distinction is critical when evaluating immune markers in TB, as certain responses may indicate exposure and not necessarily confer protection against progression to active disease. Developing better vaccines is essential for TB control (World Health Organization, 2024; World Health Organization, 2025). While the Bacillus Calmette–Guérin (BCG) vaccine offers some defense against pulmonary TB, its protection is inconsistent and tends to decline over time (Abubakar et al., 2013; Martinez et al., 2022). This has spurred efforts to create new vaccines that can strengthen or supersede the immunity provided by BCG. One of the biggest challenges in advancing TB vaccine candidates is the absence of well−validated correlates of protection (CoPs)—biological markers that could reliably predict vaccine efficacy, inform iterative vaccine design, and support comparisons across different populations (Bhatt et al., 2015; Wang et al., 2024).

For decades, research on TB immunity has centered on Th1−type cellular responses, notably the production of IFN−γ, IL−2, and TNF−α by antigen−specific CD4 T cells (Desvignes and Ernst, 2009; Cavalcanti et al., 2012). IFN−γ plays a key role in activating macrophages and controlling mycobacterial growth (Desvignes and Ernst, 2009); TNF−α helps form and maintain granulomas (Algood et al., 2005; Yuk et al., 2024); and IL−2 promotes T−cell proliferation and the development of immunological memory (Ross and Cantrell, 2018; Shouse et al., 2024). Given these functions—together with the routine clinical use of interferon−γ release assays (IGRAs)—many researchers have considered antigen−specific Th1 cytokine profiles, particularly “polyfunctional” CD4 T cells that co−express IFN−γ, IL−2, and TNF−α, as biologically plausible candidates for a CoP (Lewinsohn et al., 2017).

Nevertheless, the clinical data connecting such cytokine readouts to actual protection have not been consistent (Lewinsohn et al., 2017). Some reports indicate that strong antigen−specific responses might merely reflect the degree of recent exposure or antigen load, potentially marking increased risk rather than protection (Fletcher et al., 2016; Nemes et al., 2022). Furthermore, vaccine trials have repeatedly shown a disconnect between immunogenicity and efficacy: certain candidates elicit robust Th1 cytokine responses without reducing disease or infection (Tameris et al., 2013; Ndiaye et al., 2015), while others demonstrate protection in the absence of a clear cytokine−based signature (Van Der Meeren et al., 2018; Tait et al., 2019).

To clarify these issues, we performed a systematic review and meta−analysis that evaluated antigen−specific IFN−γ, IL−2, and TNF−α as potential correlates of protection or risk. We adopted a pre−specified, two−level analytical approach: (i) a primary synthesis limited to studies that used progression to active TB disease as the clinical endpoint, and (ii) a secondary synthesis focused on sustained IGRA conversion or other endpoints. Additionally, we included a structured narrative summary of vaccine efficacy trials that reported Th1 cytokine immunogenicity data but did not provide standardized individual−level estimates linking cytokine measures to outcomes.

## 2 Methods

This systematic review and meta-analysis was conducted following the PRISMA 2020 guidelines. The review protocol was prospectively registered in PROSPERO (registration number: INPLASY202610094, INPLASY.COM), ensuring that the review methods and scope were pre-specified. If not, this should be explicitly stated as: ‘The review protocol was not prospectively registered.

### 2.1 Information sources and search strategy

We performed comprehensive literature searches in PubMed/MEDLINE, Embase, Web of Science, and the Cochrane Central Register of Controlled Trials (CENTRAL) from their inception until June 30, 2025. All databases were updated to this same cut-off date to ensure consistency across the search process. Searches were also extended to clinical trial registries, including ClinicalTrials.gov and the WHO International Clinical Trials Registry Platform (ICTRP). To ensure thorough coverage, reference lists of all eligible studies and relevant systematic reviews were manually screened for additional records.

The search strategy incorporated both controlled vocabulary (MeSH, Emtree) and free-text keywords encompassing three key domains: tuberculosis, vaccination/exposure cohorts, and antigen-specific T-cell cytokine readouts. The core search structure was: (“tuberculosis” OR “Mycobacterium tuberculosis”) AND (“vaccine” OR “vaccination” OR “trial” OR “cohort” OR “prospective”) AND (“IFN-gamma” OR “interferon-gamma” OR “IL-2” OR “interleukin-2” OR “TNF” OR “TNF-alpha” OR “polyfunctional” OR “intracellular cytokine staining” OR “ELISpot”) AND (“correlate*” OR “risk” OR “protection” OR “progression” OR “incident TB” OR “IGRA conversion” OR “QuantiFERON”). Complete search strings for each database are provided in **Supplementary Table S1**.

### 2.2 Eligibility criteria

Studies were included if they met the following criteria: (1) Population: Human participants of any age, including vaccinated cohorts (BCG or investigational TB vaccines) or longitudinal cohorts with documented exposure to or infection with M. tuberculosis. (2) Exposure/Biomarker: Measurement of antigen-specific IFN-γ, IL-2, and/or TNF-α responses using validated assays such as ELISpot, intracellular cytokine staining (ICS) with flow cytometry, whole-blood stimulation assays, or equivalent methods, with explicit specification of the stimulating antigen (e.g., PPD, Ag85A, ESAT-6/CFP-10, M72). (3) Study Design: Randomized controlled trials (RCTs) with immunology substudies, prospective cohort studies, or nested case-control studies. (4) Outcomes: The primary outcome was progression to microbiologically or clinically confirmed active TB disease. Secondary outcomes included sustained IGRA conversion or other trial-defined infection endpoints, such as persistent QuantiFERON positivity. (5) Data Availability: Studies must have reported sufficient data—such as hazard ratios (HR), odds ratios (OR), risk ratios (RR), or group-level counts enabling their calculation—to assess the association between cytokine measures and clinical outcomes. Narrative conclusions on such associations from prospective analyses were also considered.

We excluded animal studies, purely cross-sectional studies without longitudinal outcome data, studies reporting only immunologic correlations without linkage to disease or infection endpoints (unless used for contextual narrative synthesis), and studies lacking adequate details on antigen stimulation protocols or cytokine measurement methodology.

### 2.3 Study selection and data extraction

Study selection was performed independently by two reviewers, who first screened titles and abstracts, followed by full-text assessment. Any disagreements were resolved through discussion or, if necessary, by a third reviewer. Data were extracted using a standardized, pilot-tested form. The extraction process was carried out in duplicate by two independent reviewers, with discrepancies reconciled by consensus.

Extracted data items included: study identifiers (author, year, setting); study design; population demographics and clinical context; vaccine or exposure details; specific antigens used; assay platform; timepoints of immunologic measurement; definitions of cytokine-positive or polyfunctional T cells (including gating strategies where available); outcome definitions; reported effect estimates (HR/OR/RR) with covariate adjustments; and key narrative conclusions when direct effect estimates were unavailable.

### 2.4 Risk of bias assessment

For randomized controlled trials, we employed a domain-based assessment aligned with the Cochrane Risk of Bias tool (RoB 2), evaluating randomization, deviations from intended interventions, missing outcome data, outcome measurement, and selective reporting. For observational studies (cohort and nested case-control designs), we used a structured approach analogous to ROBINS-I or the Newcastle-Ottawa Scale, focusing on confounding control, participant selection, exposure measurement, outcome ascertainment, missing data, and selective reporting. Two reviewers independently conducted risk-of-bias assessments, with final judgments reached by consensus.

### 2.5 Statistical analysis

The principal summary measures were odds ratios (ORs) for dichotomous outcomes and mean differences (MDs) for continuous cytokine levels, each reported with 95% confidence intervals. It is important to note that the thresholds for dichotomizing cytokine responses (e.g., positive vs. negative IFN-γ or IL-2 responses) were defined in each individual study based on the assay platform and methodological conventions. These thresholds were not standardized across all studies, and variability in their application could influence the comparability of binary results. As such, comparisons across studies must consider these potential differences in dichotomization criteria.

We conducted random-effects meta-analyses when at least three clinically comparable studies assessed a similar cytokine construct and reported compatible effect measures. Random-effects models were fit using a restricted maximum likelihood (REML) estimator with Hartung–Knapp–Sidik–Jonkman adjustment for uncertainty in the pooled estimate. Statistical heterogeneity was summarized using the I² statistic.

To evaluate robustness and explore heterogeneity for the sustained IGRA conversion endpoint, we performed: (i) a leave-one-out influence analysis for the pooled IFN-γ association; (ii) subgroup meta-analysis by study design/population categories; and (iii) graphical heterogeneity/outlier diagnostics using Baujat and radial (Galbraith) plots. Small-study effects/publication bias were assessed descriptively by funnel plot inspection, recognizing that the number of contributing studies was limited and no formal asymmetry tests were emphasized.

Given heterogeneity in antigens, assay platforms, timepoints, and reporting metrics, outcomes not suitable for quantitative pooling were summarized using a structured narrative synthesis emphasizing direction, consistency, and clinical context (e.g., disease progression vs. infection endpoints; vaccine efficacy vs. immunogenicity).

## 3 Results

### 3.1 Study selection

**Figure 1:**
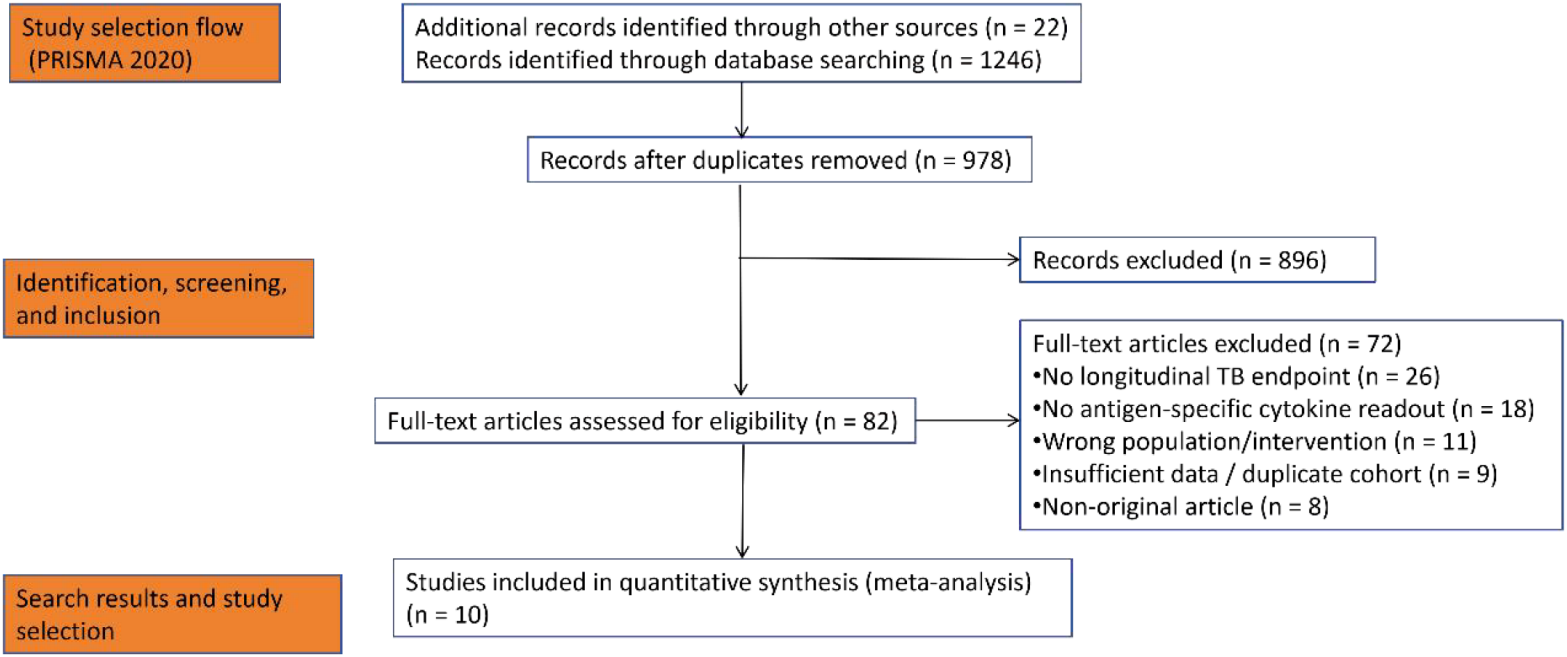
PRISMA 2020 flow diagram.

Database searches identified 1, 268 records. After removing duplicates, 978 unique records were screened based on titles and abstracts. Of these, 82 full-text articles were assessed for eligibility, resulting in the inclusion of 10 studies that met all predefined criteria. A PRISMA 2020 flow diagram detailing the selection process is presented in **Figure 1**.

### 3.2 Characteristics of included studies

The ten included studies encompassed a range of designs and populations: two prospective infant cohorts evaluating post-BCG cytokine profiles in relation to subsequent TB disease; two prevention-of-infection randomized trials using sustained IGRA conversion as their primary endpoint (assessing H4:IC31 and BCG revaccination, including a later BCG revaccination trial); three vaccine efficacy RCTs (MVA85A in BCG-vaccinated infants, MVA85A in adults living with HIV-1, and M72/AS01E in adults); one exposure/cohort study focused on IFN-γ–independent immune signatures of *M. tuberculosis* exposure; and two contextual immunology studies. The latter two—one examining the relationship between polyfunctional PPD-specific T-cell frequencies and QuantiFERON magnitude, and another investigating trained-immunity pathways following adult BCG revaccination—were retained to aid interpretation but were not included in quantitative endpoint syntheses.

**Figure 2:**
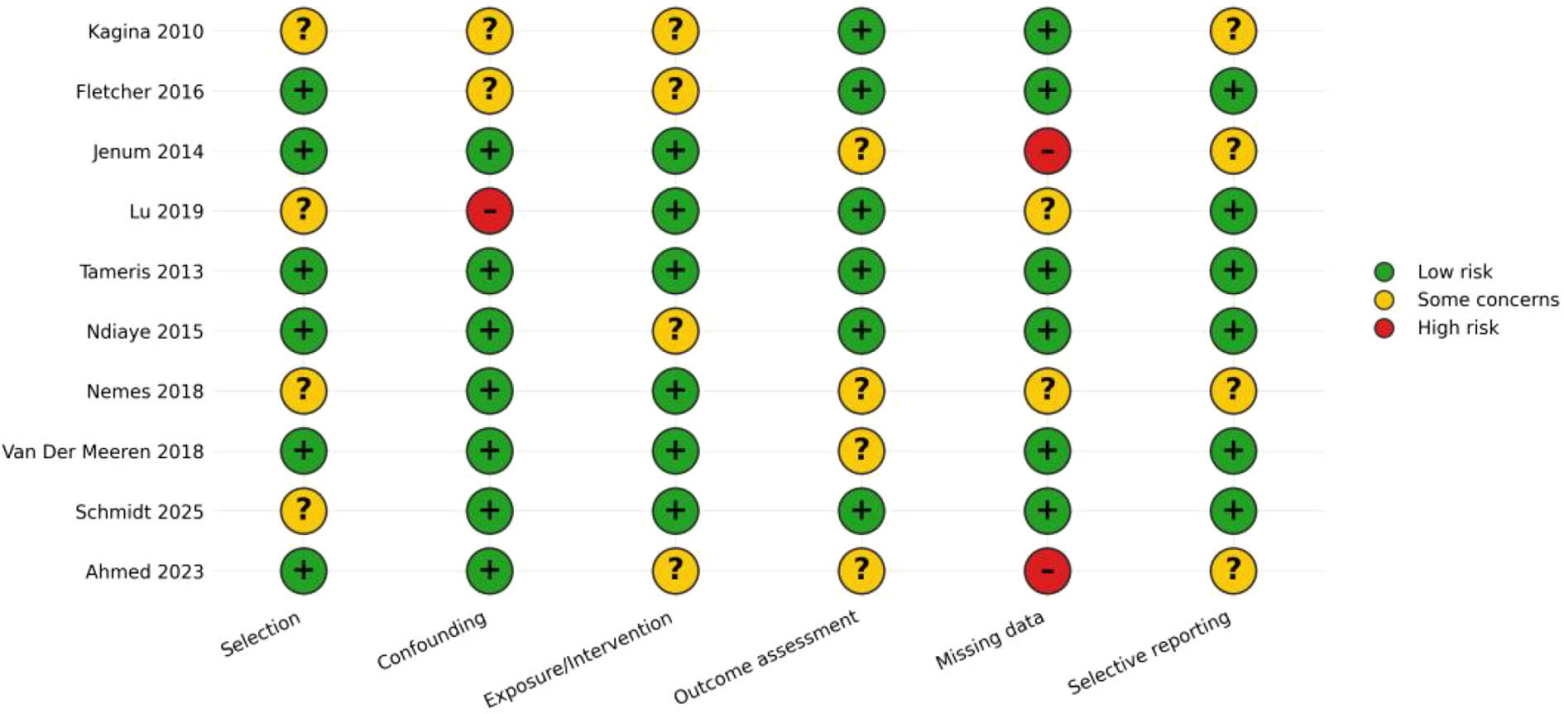
Risk of bias traffic-light plot.

**Figure 3:**
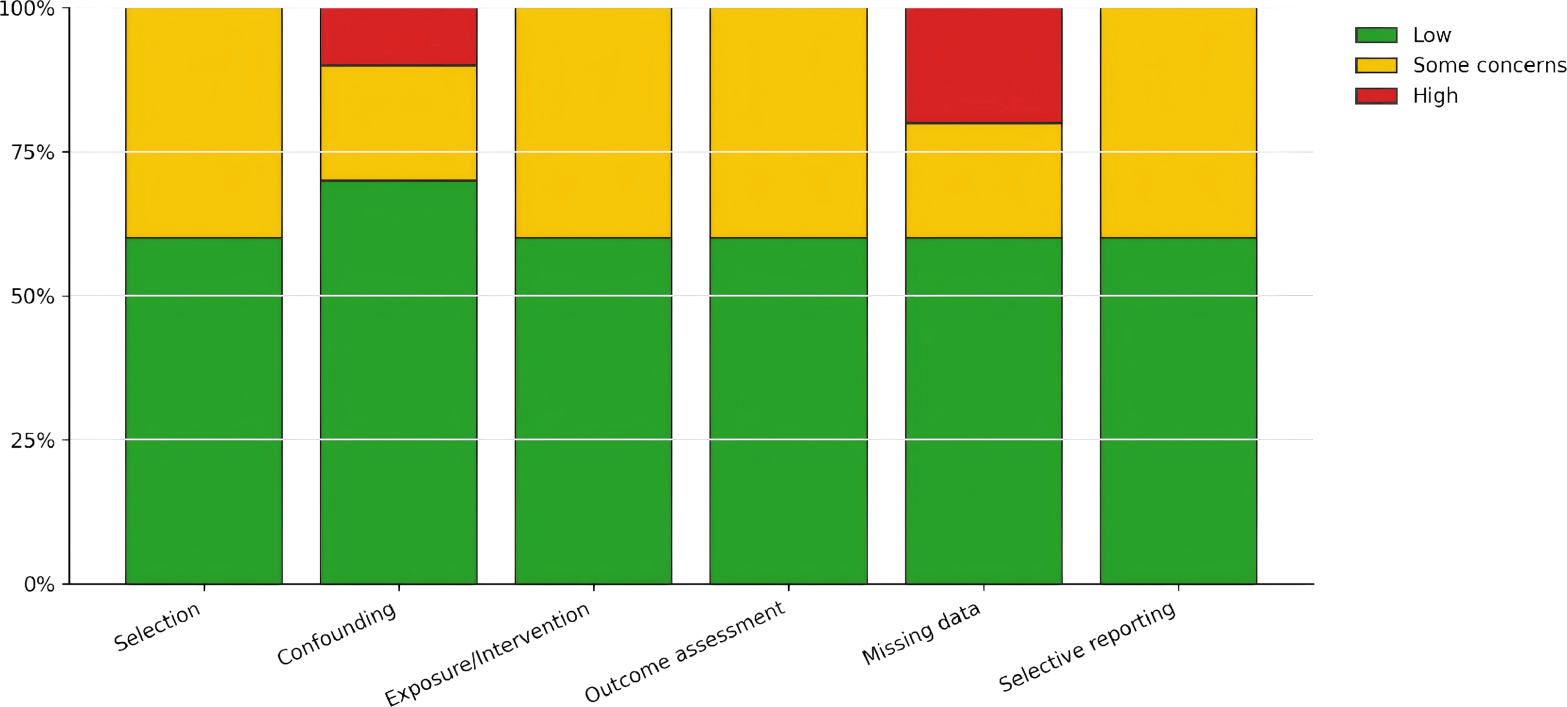
Risk of bias summary by domain.

Risk-of-bias assessments for individual studies are displayed in **Figure 2**, with a domain-level summary provided in **Figure 3**. Most studies were rated as having a low risk of bias across most domains (typically 6 out of 10 studies per domain). The remaining studies were largely judged as having “some concerns” (commonly 2–4 studies per domain, depending on the domain). High-risk ratings were infrequent and primarily pertained to missing data (2 studies) and confounding (1 study), indicating that incomplete outcome data and residual confounding represent the principal threats to internal validity within this evidence base. Key characteristics of the included studies—including antigens used (e.g., PPD, Ag85A, M72), assay platforms (e.g., ICS, ELISpot, whole-blood), and immunology sampling timepoints—are summarized in **Table 1**.

**Table 1:** Key characteristics of included studies evaluating cellular immune markers and TB-related endpoints.

Study (year)	Design/setting	Population	Intervention/exposure	Primary TB-related endpoint used in this review	Antigen (s) for immune readouts	Assay platform (s)	Key immunology timepoint(s)
Kagina et al. (2010)	Prospective infant cohort after routine neonatal BCG; South Africa	Newborns vaccinated with BCG at birth	BCG (routine)	Culture-confirmed TB disease during follow-up (protection analysis)	Whole BCG stimulation	Whole-blood stimulation followed by intracellular cytokine staining (flow cytometry)	10 weeks of age (post-BCG)
Fletcher et al. (2016)	Prospective infant study/immune-correlate-of-risk analysis; South Africa	BCG-vaccinated infants	Observational (post-BCG immunity; correlate-of-risk)	Incident TB disease (risk endpoint; not vaccine efficacy per se)	BCG and Ag85A (trial-related immunology)	Whole-blood intracellular cytokine staining and immune-phenotyping (activation markers); IFN-γ ELISpot also reported	Baseline and 28 days after immunology sampling schedule used in the parent trial context
Jenum et al. (2014)	Prospective adolescent cohort; India	Healthy Indian adolescents	Natural exposure (no vaccine efficacy endpoint)	QuantiFERON-TB Gold In-Tube response magnitude (contextual; not clinical endpoint synthesis)	PPD-specific stimulation	Flow cytometry for polyfunctional CD4 T cells (IFN-γ/IL-2/TNF-α) (reported as PPD-specific CD4+CD45RO+ cytokine+ frequencies)	Prospective sampling as reported (used to relate polyfunctionality to QFT magnitude)
Lu et al. (2019)	Exposure/cohort-based immunology study; multi-cohort	Individuals with varying M. tuberculosis exposure	Natural exposure	Exposure classification/exposure-associated immune signatures (IFN-γ–independent focus; contextual)	Multiple M. tuberculosis–related stimuli/antigens (study-specific; systems profiling)	Multi-parameter immune profiling (systems serology/cellular and other IFN-γ–independent markers; study-specific)	Cross-sectional/cohort sampling as defined in the study
Tameris et al. (2013)	Randomized, placebo-controlled phase 2b trial; South Africa	BCG-vaccinated infants	MVA85A vs placebo	TB disease (efficacy endpoint)	Vaccine antigen Ag85A (and TB-related stimuli in immunology substudies)	Trial immunogenicity included T-cell readouts (Ag85A-specific responses reported; platform details in full paper)	Post-vaccination immunology timepoints per trial schedule (reported in full paper)
Ndiaye et al. (2015)	Randomized, placebo-controlled phase 2 trial; Africa	HIV-1–infected adults	MVA85A vs placebo	TB disease (efficacy endpoint)	Vaccine antigen Ag85A (and TB-related stimuli in immunology substudies)	Trial immunogenicity included T-cell readouts (Ag85A-specific responses reported; platform details in full paper)	Post-vaccination immunology timepoints per trial schedule (reported in full paper)
Nemes et al. (2018)	Randomized, placebo-controlled prevention-of-infection trial; South Africa	Healthy adolescents	H4:IC31 vs BCG revaccination vs placebo	Initial QFT conversion; sustained QFT conversion (infection endpoints)	Ag85B+TB10.4 peptide pools (H4 components) and whole BCG	PBMC stimulation followed by intracellular cytokine staining with flow cytometry	Baseline (day 0) and day 70
Van Der Meeren et al. (2018)	Randomized, double-blind, placebo-controlled phase 2b efficacy trial; Kenya/South Africa/Zambia	IGRA-positive, HIV-negative adults (18–50y)	M72/AS01E vs placebo	Bacteriologically confirmed pulmonary TB disease (PMC)	M72 antigen (Mtb32A+Mtb39A fusion) (PMC)	Immunogenicity subgroup: anti-M72 IgG by ELISA (cell-mediated immune responses planned for later report) (PMC)	Subgroup blood draws: pre-dose 1, 1 month post-dose 2, then annually to year 3 (PMC)
Schmidt et al. (2025)	Randomized, double-blind, placebo-controlled phase 2b prevention-of-infection trial; South Africa	QFT-negative, HIV-negative adolescents	BCG revaccination vs placebo	Sustained QFT conversion (primary)	Not specified in abstract (exploratory immunogenicity reported as Th1 CD4)	Exploratory immunogenicity: induction of cytokine-positive type 1 helper CD4 T cells (platform not detailed in abstract)	Follow-up median 30 months; QFT schedule included an early post-vaccination exclusion window (day ~71)
Ahmed et al. (2023)	Adult BCG revaccination immunology study; mechanistic	Adults (BCG revaccination)	BCG revaccination (trained immunity focus)	Immunology-focused (no clinical TB endpoint synthesis)	Ag85A peptide pools; whole BCG; ESAT-6/CFP-10	Whole-blood stimulation (12h) with intracellular cytokine staining/flow cytometry readouts	Day 0 (pre) and ~day 28 (post-revaccination)

BCG, bacillus Calmette–Guérin; ICS, intracellular cytokine staining; QFT, QuantiFERON-TB Gold (IGRA); PPD, purified protein derivative; ELISpot, enzyme-linked immunospot; ELISA, enzyme-linked immunosorbent assay; TB, tuberculosis.

### 3.3 Primary analysis: Th1 cytokines and risk of active TB disease progression

**Figure 4:**
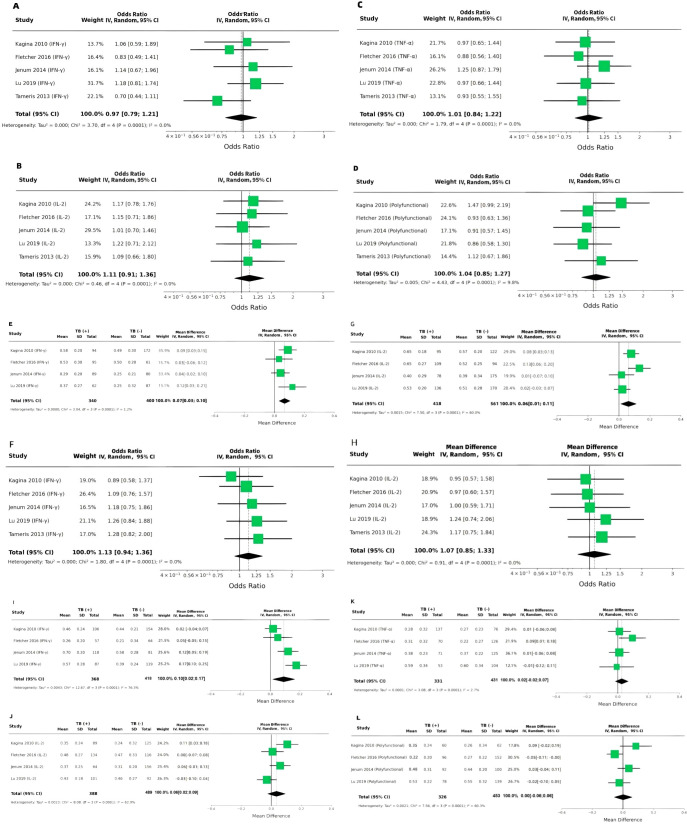
Antigen-specific Th1 cytokine markers in relation to progression to active TB disease and sustained IGRA conversion (random-effects metaanalyses). All analyses use inverse-variance random-effects models with 95% confidence intervals (CI) for each pooled estimate. Horizontal lines represent study-specific 95% CIs, squares represent study point estimates (size proportional to study weight), and diamonds represent pooled estimates. Heterogeneity is quantified using I^2^ (reported within each panel). Across sustained IGRA conversion analyses, heterogeneity was low for IFN-g (I^2^ ≈ 0%) and moderate for IL-2 continuous measures (I^2^ ≈ 60%). For progression to active TB disease, heterogeneity was low for binary markers (I^2^ ≈ 0% across most panels), while substantial heterogeneity was observed for continuous IFN-g (I^2^ ≈ 76%) and IL-2 (I^2^ ≈ 63%). **(A)** IFN-γ as a binary marker for progression to active TB disease, expressed as odds ratio (OR). **(B)** IL-2 as a binary marker for progression to active TB disease, expressed as OR. **(C)** TNF-α as a binary marker for progression to active TB disease, expressed as OR. **(D)** Polyfunctional T-cell responses as a binary marker for progression to active TB disease, expressed as OR. **(E)** IFN-γ as a continuous marker for sustained IGRA conversion, expressed as mean difference (MD). **(F)** IFN-γ as a binary marker for sustained IGRA conversion, expressed as OR. **(G)** IL-2 as a continuous marker for sustained IGRA conversion, expressed as MD. **(H)** IL-2 as a binary marker for sustained IGRA conversion, expressed as OR. **(I)** IFN-γ as a continuous marker for progression to active TB disease, expressed as MD. **(J)** IL-2 as a continuous marker for progression to active TB disease, expressed as MD. **(K)** TNF-α as a continuous marker for progression to active TB disease, expressed as MD. **(L)** Polyfunctional T-cell responses as a continuous marker for progression to active TB disease, expressed as MD.

In the primary analysis, which used progression to active TB disease as the clinical endpoint, associations between various antigen-specific Th1 immune markers and disease risk were generally inconsistent and close to the null. For IFN-γ, the random-effects pooled OR was 0.97 (95% CI 0.79–1.21), with negligible between-study heterogeneity (I² = 0%) (**Figure 4A**). Similarly, the pooled OR for IL-2 was 1.11 (95% CI 0.91–1.36; I² = 0%) (**Figure 4B**), and for TNF-α it was 1.01 (95% CI 0.84–1.22; I² = 0%) (**Figure 4C**). Analysis of polyfunctional T-cell responses (e.g., co-expressing IFN-γ, IL-2, and TNF-α) also yielded a pooled estimate near the null (OR = 1.04, 95% CI 0.85–1.27), with low heterogeneity (I² = 9.8%) (**Figure 4D**).

### 3.4 Secondary analysis: IFN-γ and IL-2 as potential indicators of exposure/antigen load

In the secondary analysis using sustained IGRA conversion as the endpoint, continuous measures of antigen-stimulated IFN-γ responses were slightly elevated among IGRA converters. The pooled MD was 0.07 (95% CI 0.03–0.10), with very low heterogeneity (I^2^ = 1.2%) (**Figure 4E**). While the effect sizes observed are modest, these small differences in cytokine levels may still have biological relevance. Even slight elevations in cytokine responses, such as IFN-γ and IL-2, could reflect nuanced variations in immune activation associated with recent antigen exposure or infection status, particularly in the context of ongoing immune surveillance. When analyzed as a binary measure, IFN-γ responses showed a similar, albeit modest, trend toward association with infection (pooled OR = 1.13, 95% CI 0.94–1.36; I^2^ = 0%) (**Figure 4F**). For IL-2, continuous measures were also marginally higher in converters (pooled MD = 0.06, 95% CI 0.01–0.11), though with moderate heterogeneity (I^2^ = 60.0%) (**Figure 4G**). The binary analysis for IL-2 yielded a pooled OR of 1.07 (95% CI 0.85–1.33; I^2^ = 0%) (**Figure 4H**).

### 3.5 Primary analysis: continuous Th1 markers and disease risk

Analyzed as continuous variables in relation to active TB disease progression, IFN-γ levels showed a pooled mean difference of 0.10 (95% CI 0.02–0.17), albeit with substantial heterogeneity (I^2^ = 76.3%) (**Figure 4I**). For IL-2, the pooled MD was 0.06 (95% CI 0.02–0.09), also with considerable heterogeneity (I^2^ = 62.9%) (**Figure 4J**). In contrast, the association for TNF-α was smaller (pooled MD = 0.02, 95% CI –0.02–0.07) and more consistent across studies (I^2^ = 2.7%) (**Figure 4K**). Polyfunctional T-cell responses showed a pooled MD close to zero (0.00, 95% CI –0.06–0.06), with moderate heterogeneity (I^2^ = 60.3%) (**Figure 4L**).

All analyses use inverse-variance random-effects models with 95% confidence intervals (CI) for each pooled estimate. Horizontal lines represent study-specific 95% CIs, squares represent study point estimates (size proportional to study weight), and diamonds represent pooled estimates. Heterogeneity is quantified using I² (reported within each panel). Across sustained IGRA conversion analyses, heterogeneity was low for IFN-γ (I² ≈ 0%) and moderate for IL-2 continuous measures (I² ≈ 60%). For progression to active TB disease, heterogeneity was low for binary markers (I² ≈ 0% across most panels), while substantial heterogeneity was observed for continuous IFN-γ (I² ≈ 76%) and IL-2 (I² ≈ 63%).

### 3.6 Secondary analysis: Th1 cytokines and sustained IGRA conversion (binary outcomes)

**Figure 5:**
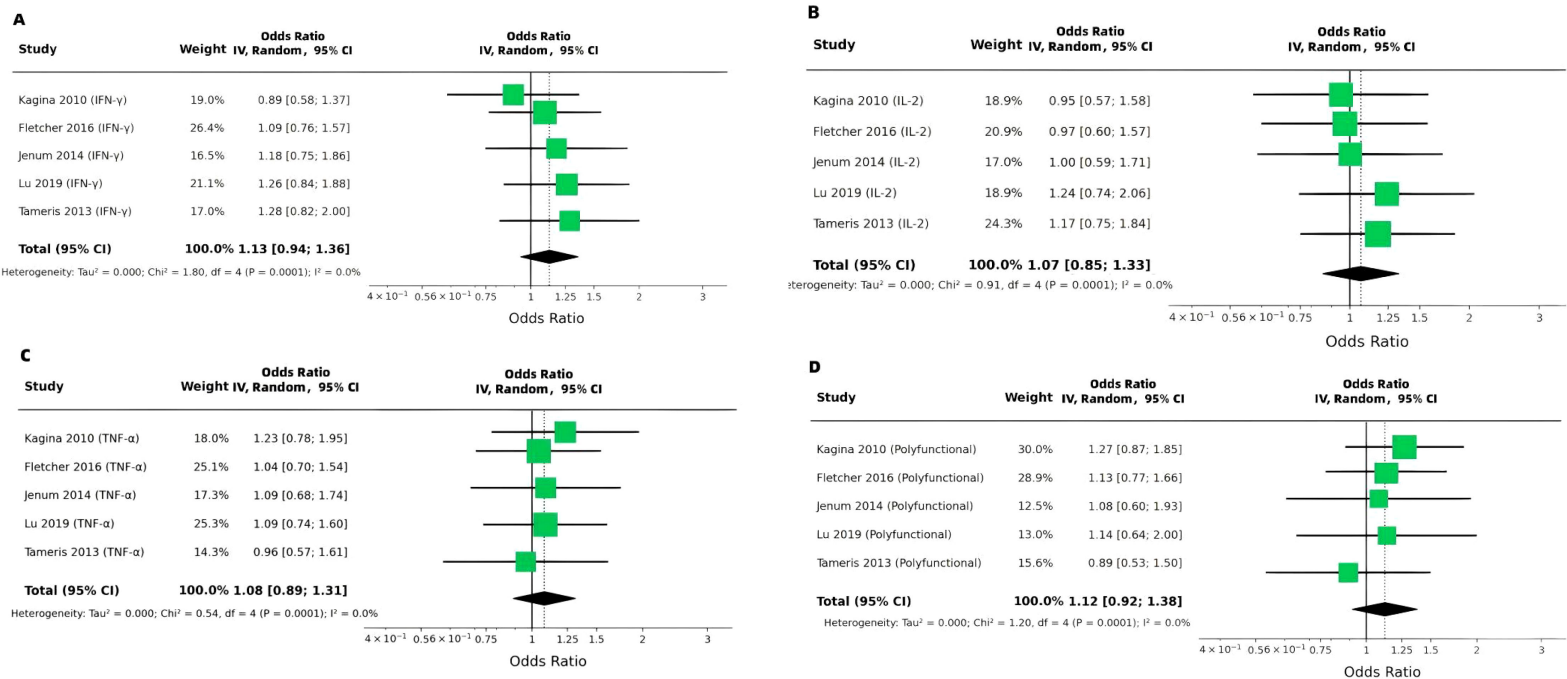
Antigen-specific Th1 cytokine markers and sustained IGRA conversion (random-effects meta-analysis, odds ratios). **(A)** IFN-γ, **(B)** IL-2, **(C)** TNF-α, and **(D)** polyfunctional T-cell responses (e.g., co-expression of IFN-γ, IL-2, and TNF-α). All analyses use inverse-variance random-effects models with 95% confidence intervals (CI). No significant association was observed for any cytokine marker, with pooled ORs near 1.0 and confidence intervals crossing the null (1.0).

When assessing binary outcomes for sustained IGRA conversion, Th1 cytokine markers collectively showed a slight trend toward positive association, though confidence intervals for most pooled estimates crossed the null. For IFN-γ, the pooled OR was 1.13 (95% CI 0.94–1.36; I² = 0%) (**Figure 5A**). The corresponding estimates for IL-2 and TNF-α were 1.07 (95% CI 0.85–1.33; I² = 0%) and 1.08 (95% CI 0.89–1.31; I² = 0%), respectively (**Figures 5B, C**). Polyfunctional responses yielded a pooled OR of 1.12 (95% CI 0.92–1.38; I² = 0%) (**Figure 5D**).

### 3.7 Secondary analysis: sustained IGRA conversion (continuous immune markers)

Continuous cytokine readouts (reported as antigen-stimulated cytokine concentrations and/or frequencies of cytokine-positive cells) were generally higher among participants who experienced sustained IGRA conversion/infection (IGRA converters) compared with non-converters across polyfunctional responses, TNF-α, IL-2, and IFN-γ outcomes (**Supplementary Figures S1A–D**). Because included studies reported continuous outcomes on non-comparable scales (e.g., different units, background subtraction approaches, stimulation antigens, and assay platforms), we present these data as a structured supplemental synthesis rather than a single pooled estimate for each marker. Nevertheless, the direction of effect was broadly concordant across panels, and for IFN-γ and IL-2 this visual pattern aligns with the modest pooled mean differences observed in the main continuous analyses, supporting the interpretation that incremental increases in Th1 cytokine magnitude may track recent M. tuberculosis exposure and antigen load. In contrast, the largely null binary results for sustained IGRA conversion/infection may reflect information loss introduced by dichotomization and the additional between-study variability arising from non-standardized positivity thresholds, which can attenuate associations when the underlying signal is small and graded. Importantly, sustained IGRA conversion/infection is an infection/exposure endpoint; therefore, higher Th1 cytokine responses in converters should be interpreted as correlates of exposure/risk rather than correlates of protection against progression to active TB disease. These findings underscore the need for future studies to standardize continuous immune readouts (including units, antigen stimulation conditions, and analytic pipelines) to enable more quantitative cross-study synthesis and to test whether multi-marker signatures outperform single cytokines for distinguishing infection risk from disease protection.

### 3.8 Sensitivity analysis: leave-one-out influence

A leave-one-out sensitivity analysis was performed to assess the robustness of the pooled association between IFN-γ responses and sustained IGRA conversion. Sequentially removing each study did not materially alter the overall random-effects summary estimate (pooled OR = 1.13, 95% CI 0.94–1.36). All leave-one-out confidence intervals overlapped with the main pooled estimate, indicating that the observed association was not driven by any single outlier study but represented a consistent, modest signal across the available evidence (**Supplementary Figure S2**).

### 3.9 Subgroup analysis (IFN-γ and sustained IGRA conversion)

**Figure 6:**
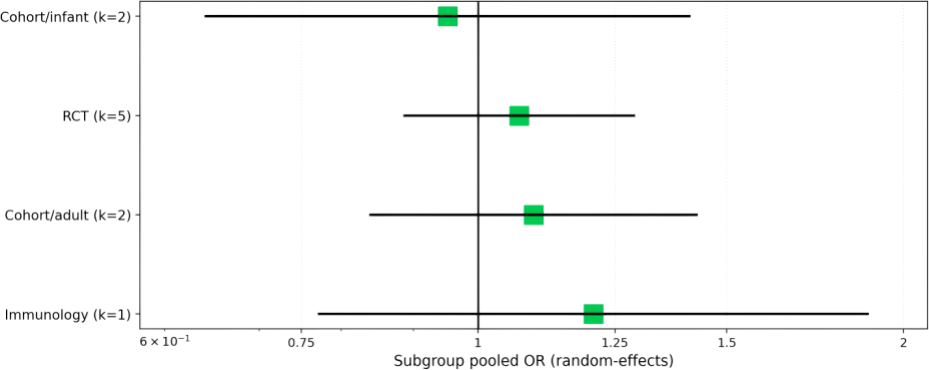
Subgroup meta-analysis: IFN-γ and sustained IGRA conversion.

We conducted a subgroup meta-analysis to explore potential effect modification by study design and population. Across pre-specified subgroups (infant cohorts, RCTs, adult cohorts, and an immunology-focused subgroup), pooled odds ratios remained close to unity, with substantial overlap in 95% confidence intervals. Point estimates were slightly above 1.0 in the RCT and adult cohort subgroups, closer to the null in the infant cohort subgroup, and higher but imprecise in the immunology subgroup (which contained only one study). Overall, these analyses revealed no clear or consistent subgroup-specific differences in the association between IFN-γ responses and sustained IGRA conversion (**Figure 6**).

### 3.10 Baujat plot (heterogeneity contribution vs. influence)

To identify studies that disproportionately contributed to overall heterogeneity and influenced the pooled effect estimate, we constructed a Baujat plot (**Supplementary Figure S3**). The plot displays each study’s contribution to total heterogeneity (Q_i_, x-axis) against its influence on the pooled log odds ratio (y-axis). Studies located in the upper-right quadrant contribute substantially to both heterogeneity and the instability of the summary estimate. In our analysis, Kagina et al. (2010) and Nemes et al. (2022) appeared in this region, suggesting they are key sources of heterogeneity and influence. Schmidt et al. (2025) and Van Der Meeren et al. (2018) also showed notable influence on the pooled effect. In contrast, studies like Tameris et al. (2013) contributed minimally. The plot thus indicates that a small number of studies likely drive both the observed heterogeneity and the sensitivity of the overall conclusion.

### 3.11 Radial (galbraith) plot (standardized effect vs. precision)

**Figure 7:**
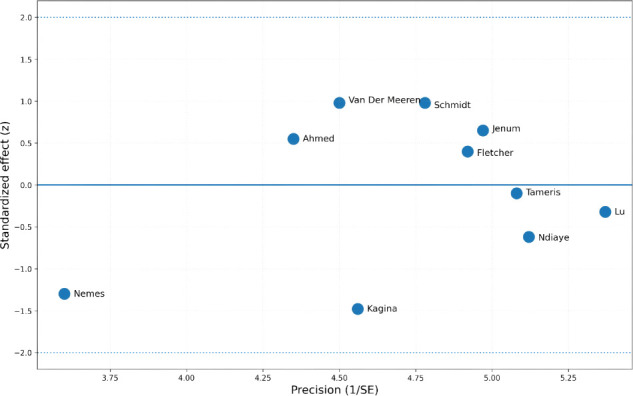
Radial (Galbraith) plot: Distribution of studies evaluating IFN-γ in relation to sustained IGRA conversion.

A Radial (Galbraith) plot was used to visually assess between-study heterogeneity and identify potential outliers (**Figure 7**). The plot displays the inverse of the standard error (precision) on the x-axis against the standardized effect (Z-score) on the y-axis. Most study points clustered near Z = 0, indicating general consistency in effect direction. However, a few studies, notably Nemes et al. (2022) and Kagina et al. (2010), showed greater vertical deviation, suggesting their effect estimates differ somewhat from the overall trend and may contribute to heterogeneity. Studies with higher precision, such as Lu et al. (2019) and Fletcher et al. (2016), are positioned toward the right of the plot and carry greater weight in the pooled estimate. The Radial plot corroborates findings from previous influence and heterogeneity analyses, helping to pinpoint potential outliers and inform sensitivity assessments.

**Figure 8:**
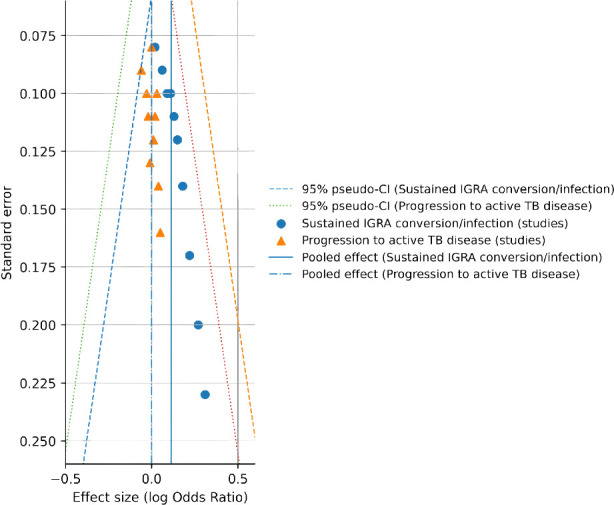
Funnel plots assessing publication bias and small-study effects for sustained IGRA conversion and progression to active TB disease.

For the outcome of sustained IGRA conversion, funnel plot inspection revealed a degree of asymmetry that may suggest small-study effects or publication bias. However, given the limited number of studies and narrow range of standard errors for this endpoint, the funnel plot should be interpreted cautiously and does not provide definitive evidence of bias (**Figure 8**).

To further aid in understanding the relationship between different endpoints and correlates, a schematic summarizing the endpoint hierarchy and their respective interpretations is provided (**Supplementary Figure S4**). This schematic illustrates how progression to active disease serves as the definitive endpoint for validating correlates of protection, while infection endpoints reflect antigen exposure and infection risk. The stratified approach ensures that these distinct types of immune responses are interpreted in their proper context.

## 4 Discussion

In this systematic review and meta-analysis, we evaluated whether antigen-specific Th1 cytokine markers—IFN-γ, IL-2, and TNF-α—serve as reliable correlates of protection (CoP) against tuberculosis, utilizing a prespecified hierarchical framework that distinguished clinically definitive from proxy endpoints (Plotkin, 2010; O’Garra et al., 2013). Our primary analysis focused on progression to active TB disease, with a secondary analysis of sustained IGRA conversion (Schrager et al., 2018). The synthesized evidence across diverse immunological contexts—spanning vaccine trials and natural exposure cohorts—reveals a consistent pattern: canonical Th1 cytokine magnitudes are not stably associated with protection against active disease (Theron et al., 2012). Instead, and informatively, these responses often align more closely with immune activation states that may signal risk, particularly in settings like infant BCG vaccination (Hatherill et al., 2020). This nuanced finding, derived from integrating heterogeneous study designs, challenges a long-held simplifying assumption and redirects the search for true CoPs.

The observed inconsistency stems from several interrelated factors (O’Garra et al., 2013). First, while IFN-γ, IL-2, and TNF-α are undeniably critical for anti-mycobacterial immunity (Desvignes and Ernst, 2009; Bhatt et al., 2015), their concentration in peripheral blood may be a more accurate measure of antigen exposure intensity or load than of sterilizing immune capacity (Moreira-Teixeira et al., 2018). High antigen-specific responses can thus act as a sensitive “readout” of recent or ongoing immunological engagement, which in high-transmission settings may paradoxically correlate with higher risk of infection or disease, rather than protection (Ewer et al., 2006). This resolves a key paradox: the same immune mechanism necessary for control can, when measured as a circulating biomarker, primarily indicate the level of threat the immune system is perceiving.

Second, the technical landscape of cytokine measurement is fraught with heterogeneity that obscures true biological signals (Janetzki et al., 2009; Britten et al., 2012). Assay outcomes are highly sensitive to variables including antigen choice (PPD vs. pathogen-specific vs. vaccine antigens), stimulation protocols, platform (ICS, ELISpot, whole-blood), gating strategies, and sampling timepoints. Even within a single platform, reporting inconsistencies—in metrics like response frequency, background subtraction, and definitions of polyfunctionality—severely undermine the comparability essential for meta-analysis and CoP validation (Britten et al., 2011).

Third, protection against TB is almost certainly multifactorial and compartmentalized (Ogongo et al., 2019; Lewinsohn and Lewinsohn, 2022). Relying on peripheral blood levels of a few cytokines overlooks the crucial qualitative and spatial dimensions of immunity. A protective response depends not only on cytokine-producing capacity but also on T-cell differentiation (e.g., towards tissue-resident memory), functional avidity, state of activation or exhaustion, the contribution of trained innate immunity, antibody functions, and, critically, the lung-localized immune microenvironment where the battle against *M. tuberculosis* is ultimately fought. Peripheral cytokine assays provide a limited, and potentially misleading, window into this complex system.

The MVA85A trials induced robust antigen-specific IFN-γ responses but failed to confer protection (Lewinsohn et al., 2017; Shouse et al., 2024), while the M72/AS01E trial demonstrated efficacy without a clear Th1 cytokine signature (Fletcher et al., 2016; Van Der Meeren et al., 2018). These findings highlight that biomarkers such as Th1 cytokine responses may be necessary components of the immune response, but they are not sufficient surrogates for clinical protection. Consequently, Th1 cytokine magnitude alone is unlikely to serve as a generalizable surrogate endpoint to predict vaccine efficacy across different platforms and populations (Callegaro and Tibaldi, 2019).

The use of sustained IGRA conversion as an infection endpoint in prevention trials is valuable for accelerating vaccine candidate screening. However, our review identifies a major structural gap: such studies rarely report the harmonized, individual-level cytokine–outcome association estimates required for meaningful meta-analysis (Callegaro and Tibaldi, 2019). This gap is not merely a statistical oversight but reflects the demanding nature of high-quality correlates research, which requires prespecified plans, adequate statistical power, standardized assays, and meticulous control of confounding (Britten et al., 2012). Without a concerted shift towards consistent reporting—including effect estimates per unit change, pre-defined categorical thresholds, and adjustment sets—the field will continue to struggle to pool evidence efficiently, even from otherwise high-quality trials. Our review employed an endpoint-stratified framework to align biomarker assessment with clinically meaningful outcomes and to avoid conflating disease protection with infection proxies.

Progression to active TB disease remains the gold-standard endpoint for validating correlates of protection, as it directly reflects the clinical relevance of immune responses. In contrast, infection endpoints, such as sustained IGRA conversion, primarily reflect antigen exposure and may preferentially identify correlates of exposure rather than true protection against disease progression. Continuous cytokine measures, in particular, may offer greater sensitivity for capturing variations in antigen load or immune activation, as they do not rely on arbitrary thresholds. However, it is important to recognize that infection endpoints, such as sustained IGRA conversion, may preferentially reflect correlates of exposure or recent infection, rather than immunity that confers protection against the development of active TB disease. In contrast, binary classifications, which dichotomize responses into positive or negative categories, might oversimplify the immune response, potentially overlooking more subtle but biologically significant variations. Thus, continuous measures might be better suited for reflecting antigen exposure or immune response intensity, particularly in studies where the underlying infection burden or antigenic exposure is heterogeneous. We deliberately incorporated studies with varied interventions (vaccines and natural exposure) to test the universality of Th1 cytokines as a CoP, acknowledging that this diversity is a source of both insight and heterogeneity. However, the evidence base for the primary disease progression endpoint remains small. Furthermore, substantial heterogeneity in assays and reporting limited our quantitative synthesis to only the most comparable subsets of data, with other findings integrated narratively. Our conclusions are therefore bounded by the published literature; unpublished data or ongoing immunology programs may hold additional insights.

Progress demands coordinated action in three areas: (i) standardized immunogenicity reporting adhering to frameworks like MIATA for antigens, platforms, and metrics (Janetzki et al., 2009; Britten et al., 2011); (ii) harmonized endpoint definitions, especially for infection proxies, to enable cross-trial comparison; and (iii) individual participant data meta-analyses (IPD-MA). IPD-MA is particularly crucial as it would allow modeling cytokine responses jointly with other covariates (e.g., activation phenotypes (Hatherill et al., 2020), transcriptomic risk signatures), across unified endpoint definitions, and could identify non-linear relationships not visible in aggregate data (Britten et al., 2012; Yuk et al., 2024). Ultimately, moving beyond single-cytokine readouts to integrate mechanistic and systems immunology approaches is essential to discover multi-parameter signatures that capture the complexity of protection (Ogongo et al., 2019; Lewinsohn and Lewinsohn, 2022).

## 5 Conclusion

Taken together, the evidence from this study supports the idea that antigen-specific IFN-γ, IL-2, and TNF-α, whether measured as individual markers or polyfunctional Th1 profiles, do not reliably serve as standalone correlates of protection against progression to active TB disease. Our primary findings indicate that while some associations were observed for markers like IFN-γ and IL-2 in the context of sustained IGRA conversion, the effect sizes were modest and point more toward correlates of antigen exposure rather than protective immunity. These findings highlight the ongoing immunogenicity-efficacy disconnect observed in TB vaccine trials and emphasize that the cytokine responses measured in peripheral blood alone are insufficient surrogates for disease protection. For future correlates discovery and TB vaccine development, harmonization of assay standards, transparent definitions of clinical endpoints, and multiparameter integration are essential to advancing more robust immune signatures for distinguishing infection risk from protection. These cytokine measures likely capture the dual realities of immune activation and antigen exposure as much as, or more than, they indicate protective capacity. The integration of evidence from both vaccine trials and natural history cohorts was pivotal in reaching this more nuanced understanding. Future TB vaccine development and correlates discovery must therefore prioritize standardized reporting, clinically meaningful endpoint stratification, and the pursuit of integrative, multi-omic, and functionally validated immune signatures in adequately powered prospective studies.

## Data Availability

The original contributions presented in the study are included in the article/**Supplementary Material**. Further inquiries can be directed to the corresponding author.

## Supplementary material

Supplementary Figure 1 Secondary analysis: continuous immune markers associated with sustained IGRA conversion. **(A)** Polyfunctional (continuous marker): IGRA+ vs IGRA−. **(B)** TNF-α (continuous marker): IGRA+ vs IGRA−. **(C)** IL-2 (continuous marker): IGRA+ vs IGRA−. **(D)** IFN-γ (continuous marker): IGRA+ vs IGRA−

Supplementary Figure 2 Sensitivity analysis: leave-one-out influence analysis for IFN-γ and sustained IGRA conversion.

Supplementary Figure 3 Baujat plot: Study-level contributions to heterogeneity and influence on the pooled effect.

Supplementary Figure 4 Endpoint Hierarchy in Tuberculosis Research.
