# Correction: Antigen-specific Th1 cytokine markers and protection against tuberculosis: a systematic review and meta-analysis stratified by progression to active disease and sustained IGRA conversion

**DOI:** 10.3389/fcimb.2026.1838266

**Published:** 2026-06-17

**Authors:** TianYu Lin, Sheng Liu, Yan-Yu Pan

**Affiliations:** 1 Fuzong Clinical Medical College of Fujian Medical University, Fuzhou, China; 2 The Second Department of Infection, 900th Hospital of PLA Joint Logistic Support Force, Fuzhou, China

**Keywords:** correlate of protection, correlate of risk, IFN-γ, IGRA conversion, IL-2, polyfunctional T cells, systematic review, TNF-α

There was a mistake in **Figure 4** as published. The corrected 4 and caption appears below.

**Figure 4:**
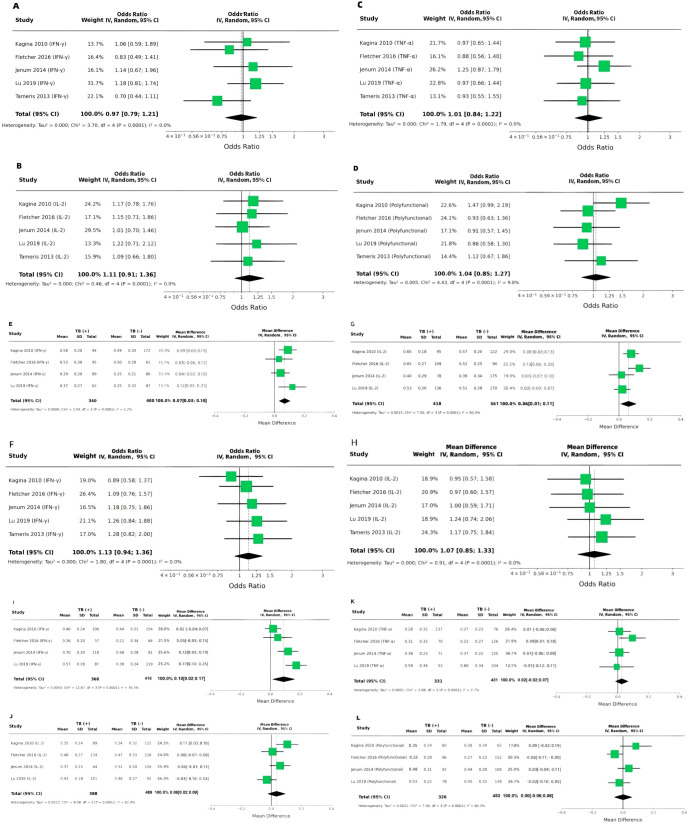
Antigen-specific Th1 cytokine markers in relation to progression to active TB disease and sustained IGRA conversion (random-effects meta-analyses). All analyses use inverse-variance random-effects models with 95% confidence intervals (CI) for each pooled estimate. Horizontal lines represent study-specific 95% CIs, squares represent study point estimates (size proportional to study weight), and diamonds represent pooled estimates. Heterogeneity is quantified using I² (reported within each panel). Across sustained IGRA conversion analyses, heterogeneity was low for IFN-γ (I² ≈ 0%) and moderate for IL-2 continuous measures (I² ≈ 60%). For progression to active TB disease, heterogeneity was low for binary markers (I² ≈ 0% across most panels), while substantial heterogeneity was observed for continuous IFN-γ (I² ≈ 76%) and IL-2 (I² ≈ 63%). **(A)** IFN-γ as a binary marker for progression to active TB disease, expressed as odds ratio (OR). **(B)** IL-2 as a binary marker for progression to active TB disease, expressed as OR. **(C)** TNF-α as a binary marker for progression to active TB disease, expressed as OR. **(D)** Polyfunctional T-cell responses as a binary marker for progression to active TB disease, expressed as OR. **(E)** IFN-γ as a continuous marker for sustained IGRA conversion, expressed as mean difference (MD). **(F)** IFN-γ as a binary marker for sustained IGRA conversion, expressed as OR. **(G)** IL-2 as a continuous marker for sustained IGRA conversion, expressed as MD. **(H)** IL-2 as a binary marker for sustained IGRA conversion, expressed as OR. **(I)** IFN-γ as a continuous marker for progression to active TB disease, expressed as MD. **(J)** IL-2 as a continuous marker for progression to active TB disease, expressed as MD. **(K)** TNF-α as a continuous marker for progression to active TB disease, expressed as MD. **(L)** Polyfunctional T-cell responses as a continuous marker for progression to active TB disease, expressed as MD.

An incorrect **Funding** statement was provided. The correct funder is 2022QH1336. The correct funding statement reads:

Startup Fund for scientific research, Fujian Medical University (Grant number: 2022QH1336).

The original version of this article has been updated.

A correction has been made to the section 3.4 *Secondary Analysis: IFN-γ and IL-2 as Potential Indicators of Exposure/Antigen Load*; 3.5 *Primary Analysis: Continuous Th1 Markers and Disease Risk*. Page 4 and 5:

3.4 *Secondary Analysis: IFN-γ and IL-2 as potential indicators of exposure/antigen load*

In the secondary analysis using sustained IGRA conversion as the endpoint, continuous measures of antigen-stimulated IFN-γ responses were slightly elevated among IGRA converters. The pooled MD was 0.07 (95% CI 0.03–0.10), with very low heterogeneity (I² = 1.2%) (**Figure 4E**). While the effect sizes observed are modest, these small differences in cytokine levels may still have biological relevance. Even slight elevations in cytokine responses, such as IFN-γ and IL-2, could reflect nuanced variations in immune activation associated with recent antigen exposure or infection status, particularly in the context of ongoing immune surveillance. When analyzed as a binary measure, IFN-γ responses showed a similar, albeit modest, trend toward association with infection (pooled OR = 1.13, 95% CI 0.94–1.36; I² = 0%) (**Figure 4F**). For IL-2, continuous measures were also marginally higher in converters (pooled MD = 0.06, 95% CI 0.01–0.11), though with moderate heterogeneity (I² = 60.0%) (**Figure 4G**). The binary analysis for IL-2 yielded a pooled OR of 1.07 (95% CI 0.85–1.33; I² = 0%) (**Figure 4H**).

3.5 **Primary Analysis: Continuous Th1 Markers and Disease Risk**

Analyzed as continuous variables in relation to active TB disease progression, IFN-γ levels showed a pooled mean difference of 0.10 (95% CI 0.02–0.17), albeit with substantial heterogeneity (I² = 76.3%) (**Figure 4I**). For IL-2, the pooled MD was 0.06 (95% CI 0.02–0.09), also with considerable heterogeneity (I² = 62.9%) (**Figure 4J**). In contrast, the association for TNF-α was smaller (pooled MD = 0.02, 95% CI −0.02–0.07) and more consistent across studies (I² = 2.7%) (**Figure 4K**). Polyfunctional T-cell responses showed a pooled MD close to zero (0.00, 95% CI −0.06–0.06), with moderate heterogeneity (I² = 60.3%) (**Figure 4L**).

The original version of this article has been updated.

