# Supplementary figures and images for: Antigen-specific Th1 cytokine markers and protection against tuberculosis: a systematic review and meta-analysis stratified by progression to active disease and sustained IGRA conversion
Source: Front Cell Infect Microbiol. 2026 Feb 20;16:1780600. doi: 10.3389/fcimb.2026.1780600 (PMC12963246; doi:10.3389/fcimb.2026.1780600)

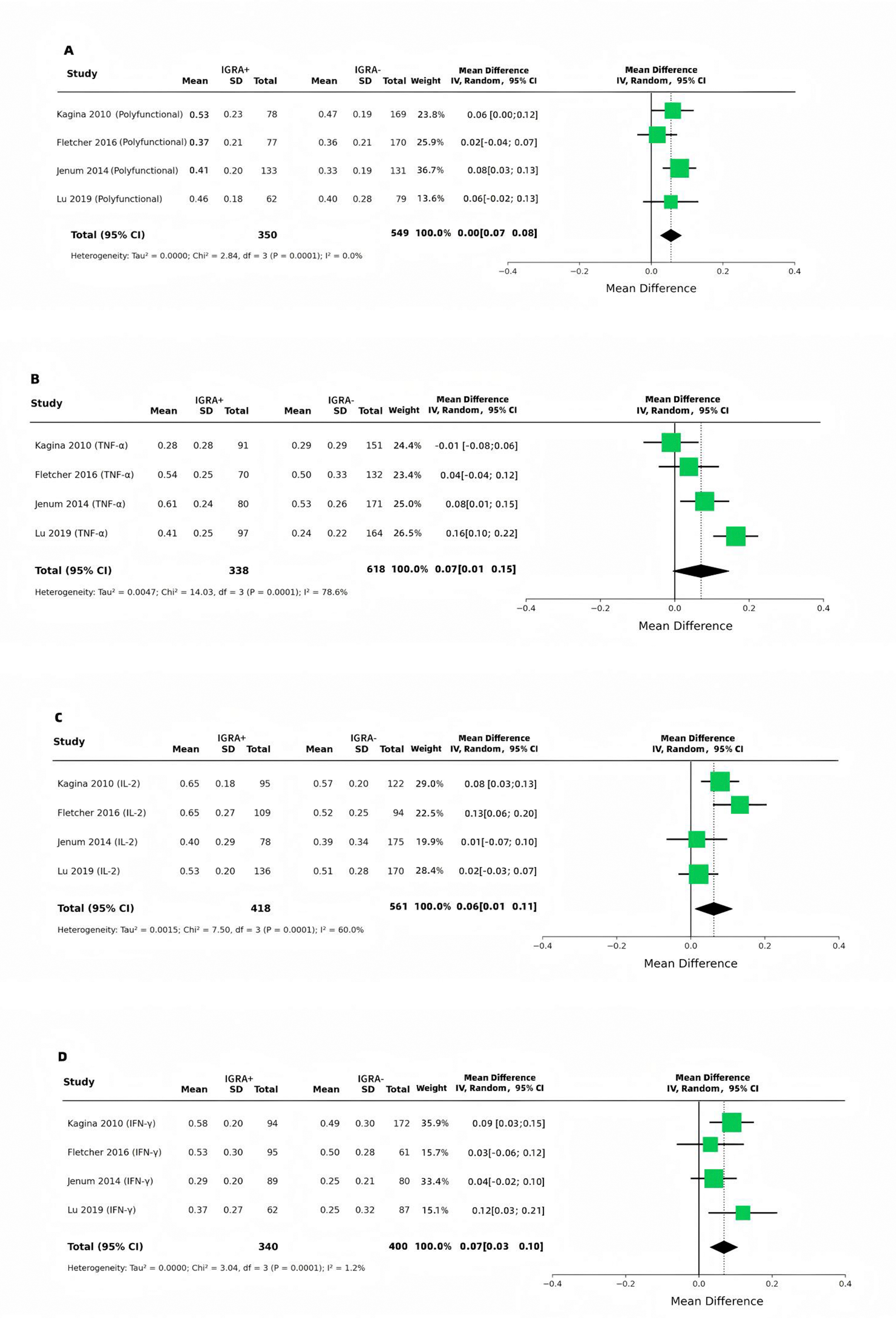

Supplement: Supplementary Figure 1 — Secondary analysis: continuous immune markers associated with sustained IGRA conversion. (A) Polyfunctional (continuous marker): IGRA+ vs IGRA−. (B) TNF-α (continuous marker): IGRA+ vs IGRA−. (C) IL-2 (continuous marker): IGRA+ vs IGRA−. (D) IFN-γ (continuous marker): IGRA+ vs IGRA− [file Image1.png]

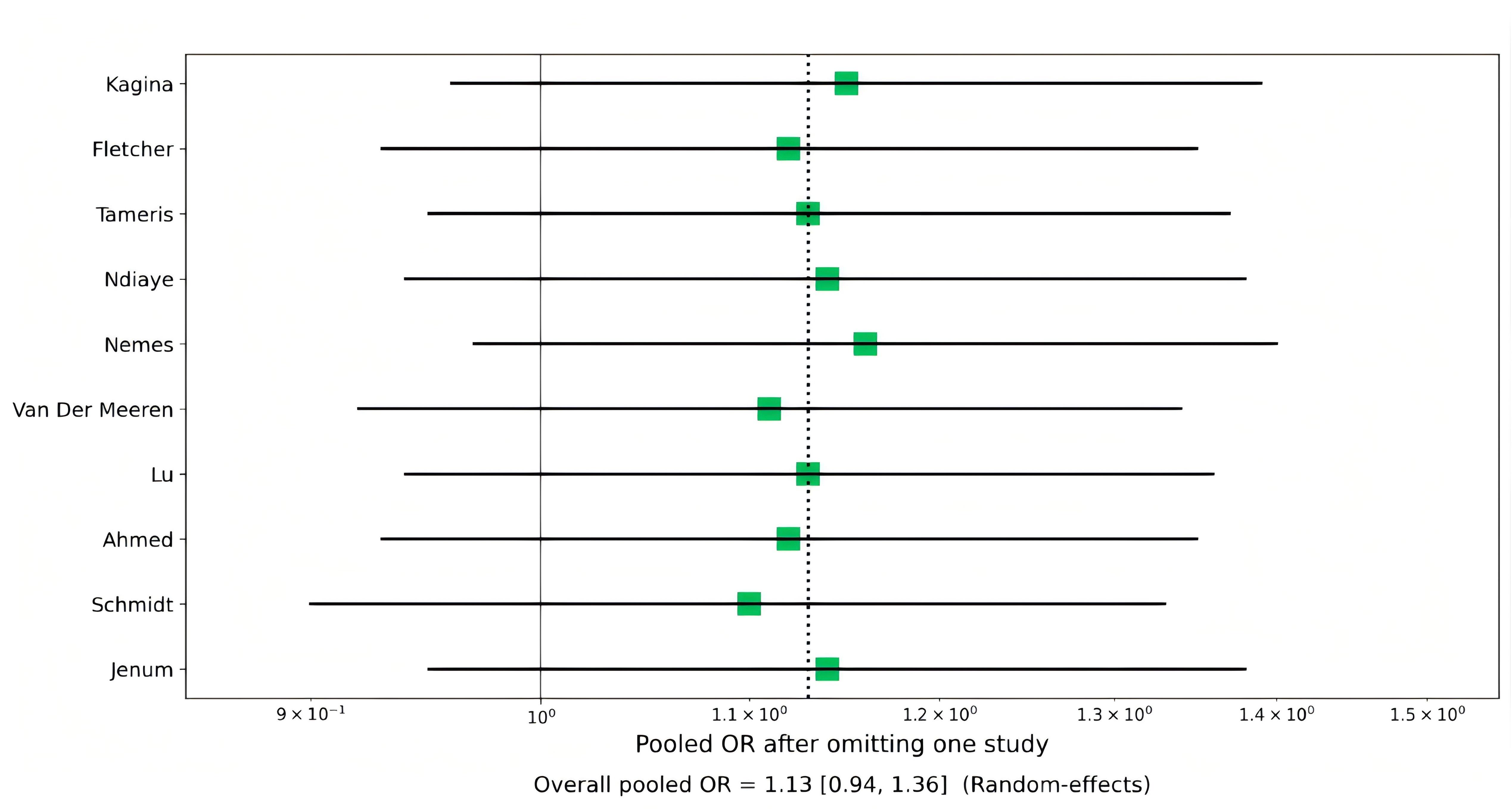

Supplement: Supplementary Figure 2 — Sensitivity analysis: leave-one-out influence analysis for IFN-γ and sustained IGRA conversion. [file Image2.jpg]

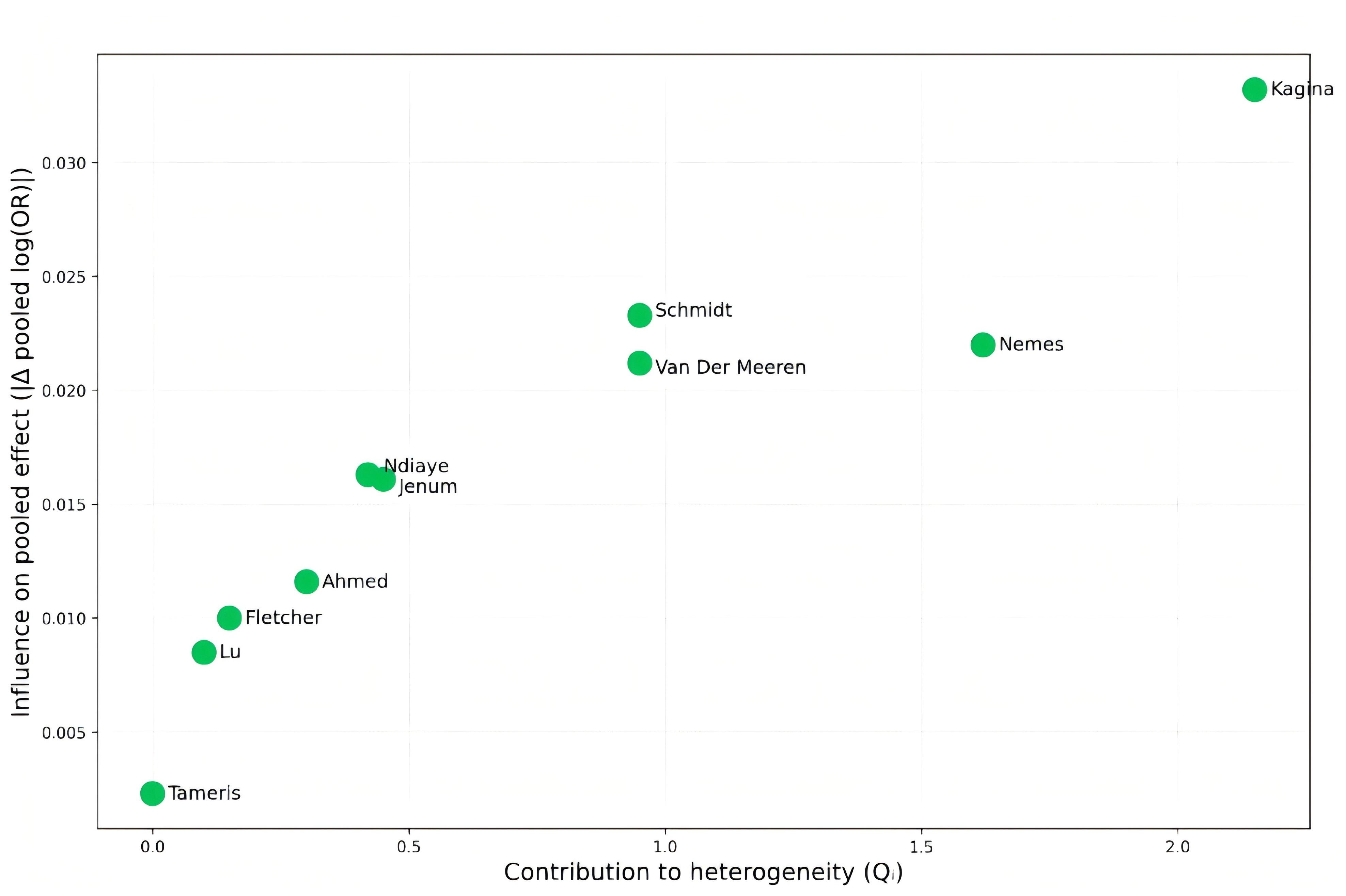

Supplement: Supplementary Figure 3 — Baujat plot: Study-level contributions to heterogeneity and influence on the pooled effect. [file Image3.jpg]

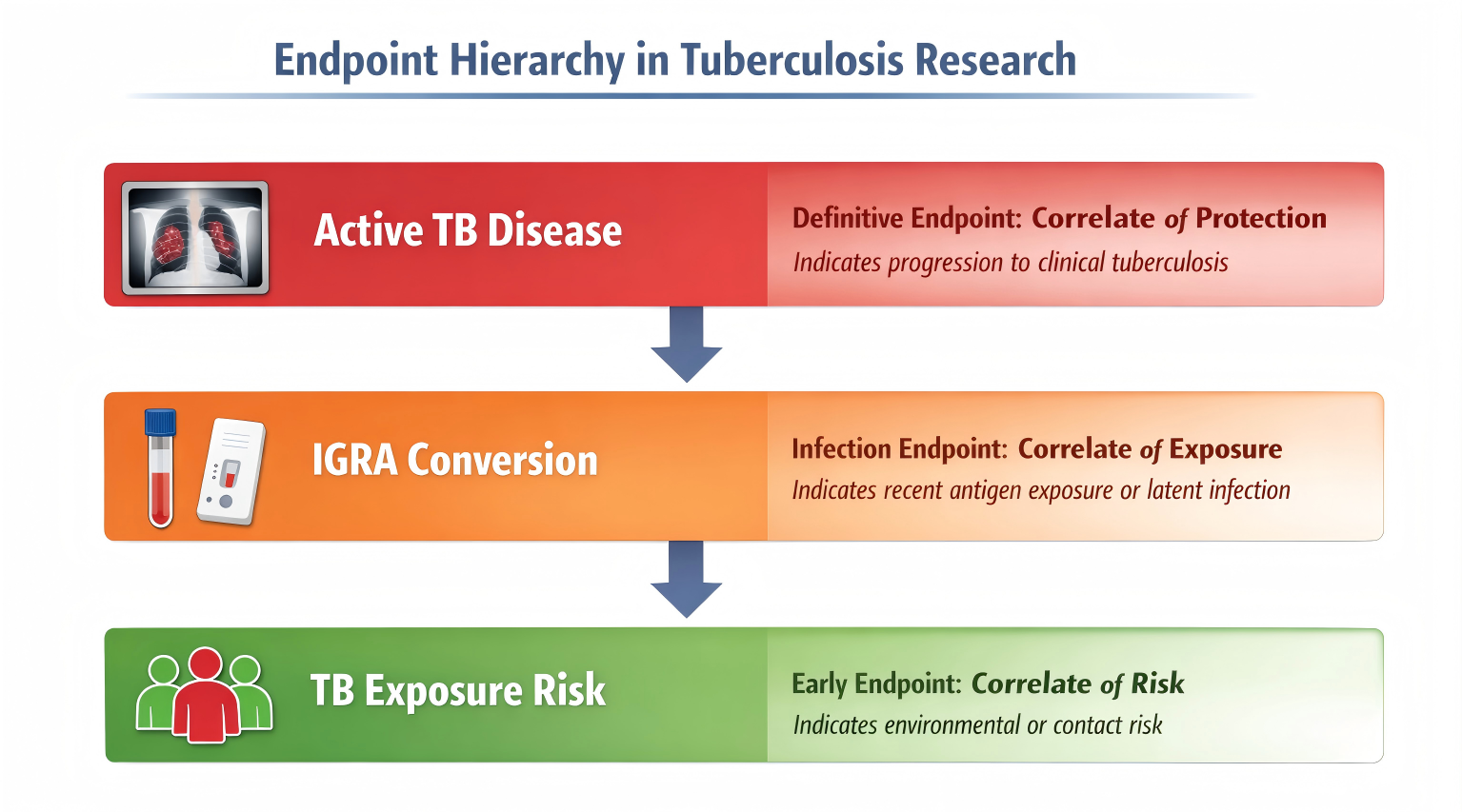

Supplement: Supplementary Figure 4 — Endpoint Hierarchy in Tuberculosis Research. [file Image4.png]
