# Supplementary material for: Antigen-specific Th1 cytokine markers and protection against tuberculosis: a systematic review and meta-analysis stratified by progression to active disease and sustained IGRA conversion
Source: Front Cell Infect Microbiol. 2026 Feb 20;16:1780600. doi: 10.3389/fcimb.2026.1780600 (PMC12963246; doi:10.3389/fcimb.2026.1780600)
Supplement: Supplementary file 5 [file Table1.docx]

# Supplementary Table S1. Full search strings used for each database

This table provides the detailed search strategies used across each electronic database. Controlled vocabulary (e.g., MeSH, Emtree) was combined with relevant free-text terms. Searches were conducted on June 30, 2025, and results were limited to human studies where applicable.

| **Database** | **Search String** |
| --- | --- |
| PubMed/MEDLINE | ("tuberculosis"[MeSH Terms] OR "Mycobacterium tuberculosis"[MeSH Terms]) AND ("vaccine"[MeSH Terms] OR "vaccination"[MeSH Terms] OR trial[tiab] OR cohort[tiab] OR prospective[tiab]) AND ("IFN-gamma"[tiab] OR "interferon-gamma"[tiab] OR "IL-2"[tiab] OR "interleukin-2"[tiab] OR "TNF"[tiab] OR "TNF-alpha"[tiab] OR "polyfunctional"[tiab] OR "intracellular cytokine staining"[tiab] OR "ELISpot"[tiab]) AND ("correlate*"[tiab] OR "risk"[tiab] OR "protection"[tiab] OR "progression"[tiab] OR "incident TB"[tiab] OR "IGRA conversion"[tiab] OR "QuantiFERON"[tiab]) |
| Embase | ('tuberculosis'/exp OR 'mycobacterium tuberculosis'/exp) AND ('vaccine'/exp OR 'vaccination'/exp OR trial:ti,ab OR cohort:ti,ab OR prospective:ti,ab) AND ('interferon gamma':ti,ab OR 'il 2':ti,ab OR 'tnf alpha':ti,ab OR 'elispot':ti,ab OR 'intracellular cytokine staining':ti,ab OR 'polyfunctional':ti,ab) AND (correlate*:ti,ab OR risk:ti,ab OR protection:ti,ab OR progression:ti,ab OR 'incident tb':ti,ab OR 'igra conversion':ti,ab OR quantiferon:ti,ab) |
| Web of Science | TS=(tuberculosis OR "Mycobacterium tuberculosis") AND TS=(vaccine OR vaccination OR trial OR cohort OR prospective) AND TS=("IFN-gamma" OR "interferon-gamma" OR "IL-2" OR "interleukin-2" OR "TNF" OR "TNF-alpha" OR polyfunctional OR "intracellular cytokine staining" OR ELISpot) AND TS=(correlate* OR risk OR protection OR progression OR "incident TB" OR "IGRA conversion" OR QuantiFERON) |
| Cochrane CENTRAL | ([mh "Tuberculosis"] OR [mh "Mycobacterium tuberculosis"]) AND ([mh "Vaccines"] OR trial OR cohort OR prospective) AND ("IFN-gamma" OR "interferon-gamma" OR "IL-2" OR "interleukin-2" OR "TNF" OR "TNF-alpha" OR polyfunctional OR "intracellular cytokine staining" OR ELISpot) AND (correlate* OR risk OR protection OR progression OR "incident TB" OR "IGRA conversion" OR QuantiFERON) |
